# *In-vitro *Antioxidant Activities of the Ethanolic Extracts of Some Contained-Allantoin Plants

**Published:** 2017

**Authors:** Zeliha Selamoglu, Cihan Dusgun, Hasan Akgul, Mehmet Fuat Gulhan

**Affiliations:** a *Department of Biotechnology, Faculty of Arts and Science, Ömer Halisdemir University, Nigde, Turkey .*; b *Department of Biology, Faculty of Arts and Science, Akdeniz University, Antalya, Turkey.*; c *Department of Medicinal and Aromatic Plants Vocational School of Technical Sciences, Aksaray University, Aksaray, Turkey.*

**Keywords:** Allantoin, plant extracts, antioxidant activity, in-vitro assays, Turkey

## Abstract

It has been investigated the *in-vitro* antioxidant properties of ethanol extracts of the contained-allantoin plants in this study. Contained-allantoined plant samples *Plantago lanceolata, Plantago major, Robinia pseudoacacia, Platanus orientalis *and *Aesculus hippocastanum *were tested at different concentrations. The antioxidant activities of plant samples were analysed by 1,1- diphenyl-2-picrylhydrazyl (DPPH) radical scavenging method, cupric reducing antioxidant capacity (CUPRAC), reducing power assay and β-carotene bleaching method. *Plantago major *plant showed the highest antioxidant capacity compared to other plant extracts in results of the *in-vitro* assays including 1,1- diphenyl-2-picrylhydrazyl (DPPH) radical scavenging method with 90.25 %, cupric reducing antioxidant capacity (CUPRAC) with 1.789 %, reducing power assay (FRAP) with 1.321 % and β-carotene bleaching method with 78.01 % in 1 mg/mL. The lowest antioxidant activity was determined in *Robinia pseudoacacia *plant. In conclusion, allantoin shows antioxidant properties and it has the positive effect on total antioxidant capacity.

## Introduction

Uric acid is an enzymatic end product of endogenous and dietary purine nucleotide metabolism and a potent antioxidant and scavenger of singlet oxygen and radicals in humans. Uric acid is converted to allantoin by enzymatic and electrochemical oxidation *in-vitro* and *in- vivo*. During increased oxidative stress, reactive oxygen species (ROS) can contribute to the formation of allantoin from uric acid. Allantoin is one from a number of uric acid oxidation products. Oxidation of urate to allantoin implies that urate is a scavenger of ROS. Allantoin, the predominant product of free radical-induced oxidation of uric acid, is efficiently excreted in the urine and has the potential as a biomarker of oxidative stress. Allantoin is the final product of purine catabolism and its chemical structure is formulated C_4_H_6_N_4_O_3 _and is also called 5-ureidohydantoin or glyoxyldiureide. It contains high levels of urea. Allantoin is a pharmacologically active compound (1-4). Allantoin is active in skin-soothing and rapid regeneration of skin cells. It removes corneocytes by loosening the intercellular kit or the desmosomes (protein bridges) that maintain the adhesion of corneocytes to each other. It then exfoliates dry and damaged cells and boosts the radiant appearance of the skin, whose surface becomes smoother and softer. Due to these properties, allantoin has been used in cosmetic industry in several forms (e.g. lotions, creams, suntan products, shampoos, lipsticks, and various aerosol preparations), as well as in topical pharmaceutical preparations for treatment of skin diseases for many years (5). Serum allantoin and/or the allantoin/uric acid ratio is also elevated in various chronic diseases and has been suggested to be a biomarker for superoxide anion-associated oxidative stress. Allantoin is significant in nitrogen metabolism for plant growth and development (6). It is a common constituent of plants being a component of the pathway of purine catabolism (7). The final two reactions of its production catalyzing the conversion of hypoxanthine to xanthine and the latter to uric acid are catalysed by the enzyme xanthine oxidoreductase, which may attain two inter-convertible forms, namely xanthine dehydrogenase or xanthine oxidase. The latter uses molecular oxygen as the electron acceptor and generates superoxide anion and other reactive oxygen products. The role of uric acid in conditions associated with oxidative stress is not entirely clear. Evidence mainly based on epidemiological studies suggests that increased serum levels of uric acid are a risk factor for some diseases where oxidative stress plays an important pathophysiological role. Also, allopurinol, a xanthine oxidoreductase inhibitor that lowers serum levels of uric acid exerts protective effects in situations associated with oxidative stress. There is increasing experimental and clinical evidence showing that uric acid has an important role *in- vivo* as an antioxidant. Unlike many antioxidants, the reaction of uric acid with an oxidant results in its stepwise degradation into a number of end products, and uric acid cannot be renewed once degraded (1-4). Generation of free radicals and ROS causes oxidative stress. Over production of ROS by endogenous or external sources, such as smoking, pollutants, inflammation, radiation, organic solvents or pesticides, causes to oxidative stress in human (8). Oxidative stress plays an important role in chronic diseases, age-related degenerative diseases, heart disease, cancer and in the aging process (9). Endogenous antioxidant system such as superoxide dismutase, hydrogen peroxide and catalase may eliminate the free radicals. Antioxidants can be found in wide various fruits and vegetables (10). 

Antioxidants have been shown to reduce the risk of chronic diseases including cancer and cardiovascular system by some scientific studies (8, 9, 11). Allantoin may have antioxidant properties (12). Five plant samples which contain different levels of allantoin, *Plantago lanceolata, Plantago major, Robinia pseudoacacia, Platanus orientalis *and *Aesculus hippocastanum *were determined. The aim of the present study is to investigate the antioxidant capacities of the ethanolic extracts of some contained-allantoin plants in Turkey.

## Expoerimental

Plant extracts

The plants were obtained from the Campus of Gaziantep University. Collected plants were dried at room temperature in a well-ventilated room and powdered with a blender to achieve a mean particle size less than 2 mm. Plant samples were weighed as 30 g and inserted into a soxhlet extractor that connected to a flask with 500 mL volum containing 250 mL of ethanol. The extraction was conducted at the boiling temperature of ethanol for 6 h. After extraction, the solvent was evaporated at its boiling point until complete removal of ethanol for determining the extract weight certainly.

DPPH free radical scavenging activity assay

The free radical-scavenging activity of plant extracts was measured using the method described by Brand-Williams *et al*. (1995), with some modiﬁcations (13). 0.06 mM solution of DPPH was prepared with methanol. Ethanol solution of the sample extracts at various concentrations (0.2-1 mg/mL) was added to 2.5 mL of 0.06 mM methanolic solution of DPPH and allowed to stand for 30 min at 25°C. The absorbance of the samples was measured at 517 nm against to blank samples. 0.1 mM solution of DPPH in methanol was used as control, whereas ascorbic acid was used as reference standard. All tests were performed with triplicate. Higher DPPH free radical scavenging activity is indicated with lower absorbance of the reaction mixture. A standard column was prepared using different concentrations of DPPH. The percent of DPPH decoloration of the samples was calculated according to the formula:

Antiradical activity=[(Ablank-Asample /Ablank)]*100

β-Carotene bleaching assay

Antioxidant activity was assessed using the β-carotene linoleate model system with a slight modification according to He, 2012 (14μμ^◦^C for 2 h. Vitamin C was used as a standard for comparison. As soon as the emulsion was added to each tube, the zero time absorbance was measured at 470 nm. Antiradical activity was calculated as follows:

Antiradical activity= [(Ablank-Asample /Ablank)]*100

Cupric reducing antioxidant capacity (CUPRAC)

The CUPRAC method was used according to Apak (2010), with some modifications (15). The CUPRAC method is based on the reduction to copper I [Cu(I)] of copper II [Cu(II)] by antioxidants. 10^-2^ M Cu(II) solution was prepared. 1mL Cu(II), Nc, and NH_4_Ac (pH: 7) buffer solutions were added to test tubes. 0.5 mL ethanol solution of the sample extracts at various concentrations (0.2-1 mg/mL) was added to the tubes. The tubes were waited 30 min. The absorbance at 450 nm (A_450_) was recorded against to a reagent blank. The molar absorptivity for each antioxidant pertaining to the CUPRAC method was calculated with the absorbance.

Determination of reducing power

The reducing powers of the extracts of *Plantago lanceolata, Plantago major, Robinia pseudoacacia, Platanus orientalis *and *Aesculus hippocastanum *plant samples were determined according to the method of Oyaizu, 1986 (16), with some modifications. Various concentrations of the plant extracts (10-100 μg/mL) were added in 2.5 mL of 0.2 M phosphate buffer (pH 6.6), 2.5 mL of 1% potassium ferricyanide solution and incubated at 50°C for 30 min. After incubation; 2.5 mL TCA (10%) was added in the reaction mixture. The content was centrifuged at 6000 rpm for 10 min. Then the absorbance of the reaction mixture was measure at 700 nm. Ascorbic acid (10-100 μg/mL) was used as positive control. The higher the absorbance of the reaction mixture is the greater the reducing power.

## Results

Plants may show various antioxidant properties in different biological systems in consequence of the presence of various substrates as well as the variable nature of products generated by the reaction system (17). The antioxidant capacities of plant extracts were assessed using four common assays, named 1,1- diphenyl-2-picrylhydrazyl (DPPH) radical scavenging method, cupric reducing antioxidant capacity (CUPRAC), reducing power assay (FRAP) and β-carotene bleaching method. 

 DPPH radical scavenging activity

DPPH is a synthetic radical, which commonly used in *in-vitro* determination of antiradical activity (18). Reults of DPPH radical scavenging activity in various concentrations of plant extracts are shown in table 1. 

**Table 1:** Free radical scavenging activity using 1,1- Diphenyl-2-picrylhydrazyl radical (DPPH)

**Concentration**	**The rate of scavenging of DPPH radical (%)**				
	0.2 mg/mL	0.4 mg/mL	0.6 mg/mL	0.8mg/mL	1 mg/mL
*P. major*	69.75	74.75	80.00	84.50	90.25
*P. lanceoalata*	50.50	67.75	72.25	77.75	84.25
*R. pseudoacacia*	43.75	65.75	72.50	76.25	78.25
*A. hippocastanum*	46.50	69.75	75.25	78.75	80.25
*P. orientalis*	44.75	58.25	68.75	78.00	80.50
Ascorbic acid	97.50	97.50	97.50	97.50	97.50

All tests were performed as triplicate and the results are showed as mean of data in the table

*Cupric reducing antioxidant capacity (CUPRAC)*

Ethanol extracts of all of the plants showed the ability reducing Cu^2+^ to Cu^+^. Also, all plant extracts showed antioxidant activities depend on concentrations. The extract of *Plantago major* displayed the highest reducing power Cu^2+^ to Cu^+^. The extract of *Robinia pseudoacacia *showed the lowest activity. Decreasing Cu^2+^ to Cu^+ ^is in order of *Plantago major*>* Platanus orientalis *>* Plantago lanceolata *>* Aesculus hippocastanum*.

The obtained data are shown in table 2.

**Table 2:** The results of CUPRAC assay

**Concentration**	**The absorbance in 450 nm of plant extracts**				
	0.2 mg/mL	0.4 mg/mL	0.6 mg/mL	0.8mg/mL	1 mg/mL
*P. Major*	1.330	1.694	1.741	1.753	1.789
*P. Lanceoalata*	1.001	1.339	1.491	1.571	1.679
*R. pseudoacacia*	0.789	0.820	0.907	1.230	1.515
*A. hippocastanum*	0.827	1.222	1.251	1.429	1.583
*P. orientalis*	0.840	1.350	1.594	1.732	1.772
Ascorbik acid	1.729	1.751	1.755	1.762	1.802

All tests were performed as triplicate and the results are showed as mean of data in the table

Reducing power

The results are shown in table 3. As a result of ferric reducing power activity, *Plantago major* had the highest activity and *Robinia pseudoacacia* displayed the lowest activity. In all plant extracts, it was observed that ferric reducing power activity was depending on concentration. Decreasing Fe^3+^ to Fe^2+ ^is in order of *Plantago major*>* Platanus orientalis *>* Plantago lanceolata *>* Aesculus hippocastanum*.

**Table 3:** The results of reducing power assay

**Concentrations**	**The absorbance in 450 nm of plant extracts**				
	0.2 mg/mL	0.4 mg/mL	0.6 mg/mL	0.8mg/mL	1 mg/mL
*P. Major*	1.209	1.243	1.269	1.287	1.321
*P. Lanceoalata*	1.149	1.198	1.218	1.231	1.255
*R. pseudoacacia*	1.112	1.600	1.174	1.204	1.229
*A. hippocastanum*	1.149	1.209	1.225	1.241	1.247
*P. orientalis*	1.194	1.214	1.248	1.271	1.281
Control	1.145				

All tests were performed as triplicate and the results are showed as mean of data in the table

*β* *-Carotene bleaching assay*

The results are shown in table 4. *Plantago major* has the highest activity and *Robinia pseudoacacia* showed the lowest activity in this assay. It was obtained that β-carotene bleaching activities of all plant extracts were depending on concentration. The antioxidant activity is observed in order of *Plantago major*>* Platanus orientalis *>* Plantago lanceolata *>* Aesculus hippocastanum*.

**Table 4:** The first inhibition of β-carotene bleaching assay (%).

**Concentrations**	**The absorbance at 490 nm of plant extracts**				
	0.2 mg/mL	0.4 mg/mL	0.6 mg/mL	0.8mg/mL	1 mg/mL
*P. Major*	42.37	43.97	44.94	45.85	46.37
*P. Lanceoalata*	43.04	44.31	45.45	46.77	48.54
*R. pseudoacacia*	41.69	42.49	42.6	42.88	43.04
*A. hippocastanum*	41.74	43.68	44.14	44.98	45.8
*P. orientalis*	36.03	39.52	43.57	44.26	46.31
Control	92.34	92.98	93.21	93.68	94.55

All tests were performed as triplicate and the results are showed as mean of data in the table

**Table 5:** The last inhibition of β-carotene bleaching assay (%).

**Concentrations**	**The absorbance in 490 nm of plant extracts**				
	0.2 mg/mL	0.4 mg/mL	0.6 mg/mL	0.8mg/mL	1 mg/mL
*P. Major*	71.56	73.5	74.35	75.54	78.01
*P. Lanceoalata*	66.65	67.16	68.02	69.73	71.90
*R. pseudoacacia*	65.39	66.19	67.16	68.82	69.85
*A. hippocastanum*	65.85	66.76	66.99	67.28	68.42
*P. orientalis*	63.91	65.96	68.13	70.30	71.50
Control	92.34	92.98	93.21	93.68	94.55

All tests were performed as triplicate and the results are showed as mean of data in the table

## Discussion

In this study, we examined the antioxidant capacities of ethanol extracts of contained-allantoin five plants by using 1,1- diphenyl-2-picrylhydrazyl (DPPH) radical scavenging method, cupric reducing antioxidant capacity (CUPRAC), reducing power assay and β-carotene bleaching method as *in-vitro*. In the results of all assays, *Plantago major* showed the highest antioxidant effect among ethanol extracts of other plant samples. *Robinia pseudoacacia* showed the lowest antioxidant activity. These results are supported with the studying antioxidant and antimicrobial properties of *Plantago major* leaves of Stanisavljević *et al*. (2008) (19). It was reported that *Plantago lanceolata* is a strong antioxidant (20). In the study, methanol extract of *Plantago major* showed antioxidant activity with 1,1- diphenyl-2-picrylhydrazyl (DPPH) radical scavenging method (21). Wilkinson and Brown reported that *Aesculus hippocastanum* showed antioxidant properties *in-vitro *(22). There have been no reports on the effects of *Robinia pseudoacacia* and *Platanus orientalis*.

In DPPH assay, the stabilization of DPPH free radicals cause the color changing of reaction solution which is measured by spectrophotometer to determine the scavenging activity of a tested sample (23). The obtained results with DPPH assay show the radical scavenging activity of examined samples as dose-dependent. All ethanolic plant extracts have DPPH radical scavenging activities. The extract of *Plantago major* showed the highest DPPH scavenging activity in comparison to other plant samples. *Plantago lanceolata, Platanus orientalis *and *Aesculus hippocastanum *showed lower DPPH inhibition. *Robinia pseudoacacia *showed the lowest DPPH inhibition. The antioxidant activity of *Plantago spp* was reported by the previous study (24). The study showed that *Plantago lanceolata* has antioxidant activity by using DPPH and FRAP method (25). 

Metal ions can cause lipid peroxidation that can produce free radicals and lipid peroxides (26). Therefore, metal chelating activity indicates the antioxidant and antiradical properties. Absorbance decreased of the reaction mixture indicates higher metal chelating ability. CUPRAC assay has been used by many researchers to determine reducing power of antioxidant compounds (15). This method is based on Cu^2+^–Cu^+^ reduction by antioxidants in the presence of neocuproine. In this assay, a higher absorbance indicates a higher cupric ions (Cu^2+^) reducing power. Our data are supported by study which determining antioxidant activity of stem and root extracts of Rhubarb (*Rheum ribes*) by CUPRAC method (27). 

Antioxidant compounds cause the reduction from ferric (Fe^3+^) form to the ferrous (Fe^2+^) form because of their reductive capabilities. Prussian blue-colored complex is formed by adding FeCl_3_ to the ferrous (Fe^2+^) form. Therefore, reduction can be determined by measuring the formation of Perl's Prussian blue at 700 nm (24). A higher absorbance indicates a higher ferric reducing power. The order of antioxidant activity is shown in order of *Plantago major *>* Platanus orientalis *>* Plantago lanceolata *>* Aesculus hippocastanum*. There is no previous study about *Plantago major *extract. The results of our study is supported with data obtained of P*lantago spp. *using FRAP method (28). 

The antioxidants can inhibit free radicals with β-carotene bleaching (29). It was obtained that β-carotene bleaching activity was depending on concentration of plant extracts. Antioxidant activity is shown in order of *Plantago major *>* Platanus orientalis *>* Plantago lanceolata *>* Aesculus hippocastanum*. Beta carotene bleaching method is used to determine the antioxidant activities of plant samples. The previous study showed that ethanolic extract of *Meconopsis quintuplinervia *has antioxidant activity and it is determined with beta carotene bleaching method (14). 

## Conclusions

This study showed that extracts of contanied-allantoin plant samples have antioxidant activities. It is considered that allantoin shows antioxidant properties and allantoin effects total antioxidant capacity positively. In addition, the results of this study will shed light on new researchs in the future and contribute to the scientific literature.
